# Dynamics of Afebrile *Plasmodium falciparum* Infections in Mozambican Men

**DOI:** 10.1093/cid/ciy219

**Published:** 2018-03-13

**Authors:** Beatriz Galatas, Helena Martí-Soler, Lidia Nhamussua, Pau Cisteró, Pedro Aide, Francisco Saute, Clara Menéndez, N Regina Rabinovich, Pedro L Alonso, Quique Bassat, Alfredo Mayor

**Affiliations:** 1 Centro de Investigação em Saúde de Manhiça, Maputo, Mozambique; 2 ISGlobal, Barcelona Center for International Health Research, Hospital Clínic—Universitat de Barcelona, Spain; 3 National Institute of Health, Ministry of Health, Maputo, Mozambique; 4 Harvard T. H. Chan School of Public Health, Boston, Massachusetts; 5 Catalan Institution for Research and Advanced Studies, Barcelona, Spain

**Keywords:** afebrile, asymptomatic, *Plasmodium falciparum*, malaria, dynamics

## Abstract

**Background:**

Afebrile *Plasmodium falciparum* infections usually remain undetected and untreated in the community and could potentially contribute to sustaining local malaria transmission in areas aiming for malaria elimination.

**Methods:**

Thirty-two men with afebrile *P. falciparum* infections detected with rapid diagnostic test (RDTs) were followed for 28 days. Kaplan-Meier estimates were computed to estimate probability of parasite positivity and of reducing parasitemia by half of its initial level by day 28. Trends of parasite densities quantified by microscopy and real-time quantitative polymerase chain reaction (qPCR) were assessed using Poisson regression models, and the microscopy-to-qPCR positivity ratio was calculated at each time point. Three survival distributions (Gompertz, Weibull, and gamma) were used to evaluate their strength of fit to the data and to predict the median lifetime of infection.

**Results:**

The cumulative probability of parasite qPCR positivity by day 28 was 81% (95% confidence interval [CI], 60.2–91.6). Geometric mean parasitemia at recruitment was 516.1 parasites/μL and fell to <100 parasites/μL by day 3, reaching 56.7 parasites/μL on day 28 (*P* < .001). The ratio of *P. falciparum*–positive samples by microscopy to qPCR decreased from 0.9 to 0.52 from recruitment to day 28. The best model fit to the data was obtained assuming a Gompertz distribution.

**Conclusions:**

Afebrile *P. falciparum* infections detectable by RDT in semi-immune adults fall and stabilize at low-density levels during the first 4 days after detection, suggesting a rapid decline of potential transmissibility in this hidden parasite reservoir.

**Clincial trials registration:**

NCT02698748

## Teaser

Afebrile *Plasmodium falciparum* infections detected by rapid diagnostic tests in semi-immune adults fall and stabilize at low density levels during the first 4 days after detection, suggesting a rapid decline of potential transmissibility.

Asymptomatic *Plasmodium falciparum* parasitemia is commonly defined as the presence of asexual parasites in the blood without symptoms or illness [1]. These infections, which can be either detectable using standard diagnostic tests such as microscopy or rapid diagnostic tests (RDTs) or submicroscopic if identified only by molecular techniques, are usually characterized by the absence of fever and can, therefore, be more specifically referred to as *afebrile* infections. Although fever-less infections may still cause other less evident symptoms with a substantial burden on the infected individual’s health [2], individuals with afebrile malaria infections usually do not seek clinical attention and therefore remain undetected by passive surveillance systems [3]. Proactive community cross-sectionals conducted to assess parasite prevalence rates have reported varying prevalence estimates of afebrile infections [4–6], indicating that these infections are highly prevalent in the community and could contribute to sustaining malaria transmission [7].

Albeit poorly understood, there are several parasite and host factors that could contribute to maintaining high community rates of afebrile malaria infections [3]. In high-endemic areas, exposed individuals develop a level of tolerance to *P. falciparum* infections into adulthood through which the pathogenesis of the infection is minimized while the parasite densities remain relatively low [8], thus leading to afebrile and submicroscopic infections. Although the relative contribution of silent parasite carriers with low parasitemia to overall transmission is still unknown [9], the high prevalence and prolonged duration [10, 11] of these infections, as well as their continuous production of gametocytes [12, 13], may imply that these infections are an important source for transmission. Additionally, such infections may pose challenges when monitoring malaria elimination in historically endemic areas where no further clinical cases are detected but where residual immunity among human hosts can still lead to the silent persistence of the parasite population [5, 14].

Several observational studies conducted in the early 1900s in malaria-endemic countries to quantify the durability of *P. falciparum* infections found that infections detected by microscopy lasted from a few weeks up to several years and that parasite densities and symptoms were reduced soon after the onset of infection [15–19]. However, the wide differences in host immunity among study participants, the limited sensitivity of microscopy, and the lack of genetic tools to identify reinfections that could occur during follow-up in areas of ongoing transmission [18, 20, 21] hinder the interpretation of these studies. More recently, the use of molecular techniques has allowed a more accurate characterization of the dynamics and duration of infection from a single parasite strain [18, 22–26], primarily showing that the longevity of infections decreased with increasing age [23, 26].

A better understanding of natural dynamics of afebrile *P. falciparum* infections is needed to design effective interventions that can successfully detect and tackle this parasite reservoir at community level [3]. Herein, parasite infections were longitudinally assessed in untreated afebrile adults from a perennially endemic region in southern Mozambique using molecular methods and mathematical models to better characterize dynamics of these silent *P. falciparum* infections and contribute to the ongoing discussion of the relevance of afebrile infections as a barrier for malaria elimination.

## METHODS

### Study Design

Adult men with afebrile *P. falciparum* infections recruited between January 2015 and June 2015 were treated with placebo and observed for 28 days in the context of a randomized, placebo-controlled clinical trial conducted in the district of Manhiça (southern Mozambique) [27]. This period coincided with the high malaria transmission season in the area, which is perennial but more marked during the rainy period (between November and April) [28]. The study aimed to assess the efficacy of chloroquine (CQ) to clear asymptomatic infections among healthy adults from the community. A placebo comparator was used to accurately account for the effect that the high levels of immunity expected in the study population would have on natural parasite clearance of low-level parasitemia infections. This analysis aimed to characterize the dynamics of afebrile infections in participants enrolled in the placebo group. Individuals who developed fever at any time during follow-up or who were only followed up for 1 day were excluded from this analysis.

### Sample Collection and Analysis

Healthy men were screened at the household level using a Histidine Rich Protein 2 (HRP2)–based rapid diagnostic test (RDT) from SD Bioline. All positive cases by RDT were subsequently confirmed by expert microscopy at the Centro de Investigação em Saúde de Manhiça (CISM). Asymptomatic malaria was defined as the absence of any proactively referred symptom of disease according to the screened individual, together with a documented axillary temperature <37.5°C. Enrolled individuals were randomly assigned to the CQ or placebo arm and followed up on days 1, 2, 3, 7, 14, 21, and 28 after enrollment. All individuals in the placebo group were treated at the end of follow-up according to national guidelines, and rescue treatment was administered to participants of either study arm that presented with clinical symptomatology during follow-up. Thick and thin Giemsa-stained blood slides and 50 µL of blood spotted onto filter papers were collected at every visit.

Slides were examined by 2 independent microscopists and considered negative if no parasites were seen after examination of 200 oil-immersion fields in a thick blood film. A third read was conducted in cases where the first 2 readings were discrepant. Parasite density was estimated using the Lambaréné method [29], which counts parasites against an assumed known blood volume. Density of *P. falciparum* was also assessed from filter paper blood-spots through real-time quantitative polymerase chain reaction (qPCR) assay targeting 18S ribosomal RNA [30]. Parasitemia was quantified by extrapolation of cycle thresholds (Ct) from a standard curve of *P. falciparum* 3D7 in vitro culture ring stage. Samples without amplification (no Ct detected) were considered negative. A negative control with no template DNA was run in all reactions.

### Data Analysis

Data from participants assigned to the placebo group were analyzed using Stata 14 (Stata Corp, College Station, TX, USA) and R Statistical Software (version 3.3.1, R Foundation for Statistical Computing, Vienna, Austria). Basic characteristics of participants at enrollment were summarized using arithmetic or geometric means and medians, as determined by the distribution of the data. Kaplan-Meier estimates were computed to measure the cumulative probabilities of parasite positivity by day 28 and of failure to reduce parasitemia levels by at least 50% of their initial value by day 28—or half-life probability. An infection was considered to be cleared at the time of the first negative observation by qPCR and when parasites were no longer observed for the subsequent follow-up observations. For infections in which parasite densities were reduced by >50% of their initial value by the end of follow-up, the last time point at which parasite densities were observed to fall below this threshold was considered as the time at which half-life was reached. Geometric mean parasite densities (GMPDs) and 95% confidence intervals (CIs) were calculated for each day of follow-up. A Poisson regression random-effect model was used to measure the variation of the parasite densities measured by qPCR at each day of follow-up. Finally, the proportions of infections that were positive by qPCR or by microscopy were calculated, and a ratio of positive microscopy to qPCR samples was estimated.

### Evaluation of Natural Infection Distributions

We evaluated the strength of fit to the data of 3 survival distributions considered by previous groups to model natural malaria infections (Gompertz, Weibull, and gamma) [31, 32] in order to provide consistent information to the ongoing discussion in this field. Kolmogorov-Smirnov test estimates were calculated to evaluate the strength of the fit of each of the distributions, and Akaike’s information criterion values were calculated for comparison of the model fits. We also attempted to estimate the median lifetime of infection and the probability of parasite positivity by day 28 using the abovementioned distributions, acknowledging the limitations of the data for this purpose, such as small sample size, short follow-up duration, or lack of ability to disentangle reinfections and recrudescence.

### Ethical Considerations

The protocol, consent forms, and questionnaires were approved by the CISM local ethics committee, the Ethics Committee of the Hospital Clínic of Barcelona (HCB/2015/0122), the National Bioethics Committee of Mozambique (CNBS; Ref. 173/CNBS/13) and the Mozambican Pharmaceutical Department (Ref./No. 4110/380/DF2014) before their implementation. All participants signed an informed consent form prior to the initiation of any study related activities. The clinical trial was registered at www.ClinicalTrials.gov (NCT02698748).

## RESULTS

This analysis used data from 32 of the 38 individuals originally enrolled in the placebo group of the clinical trial. Three individuals with only a single follow-up observation and 3 individuals who developed a fever after day 1 (n = 1) or day 3 (n = 2) of follow-up were consequently excluded from this analysis. Three individuals had missing qPCR data (on days 0, 1, and 21 respectively), and 9 skipped 1 day of follow-up (1 on day 2, 1 on day 3, 4 on day 7, and 3 on day 21) (Supplementary Table 1).

The average age of participants at enrollment was 25 years. The median body temperature was 36.4ºC, and the average body weight was 55.6 kg. The geometric mean parasite density on day 0 was 733parasites/μL as measured by microscopy and 516.1parasites/μL as measured by qPCR (Table 1).

**Table 1.** Basic Characteristics of Study Participants at Enrollment

Characteristic	Summary Statistics
Age^a^	26 (14) n = 32
Body temperature, °C^a^	36.4 (0.35) n = 32
Weight, kg^a^	55.6 (11.2) n = 32
Microscopy parasite density, p/μL^b^	733.0 (1088.0) n = 28
qPCR parasite density, p/μL^b^	516.1 (872.9) n = 31

Abbreviation: qPCR, quantitative polymerase chain-reaction.

^a^Statistics are given as arithmetic mean (SD).

^b^Statistics are given as geometric mean (SD).

**Figure 1.**
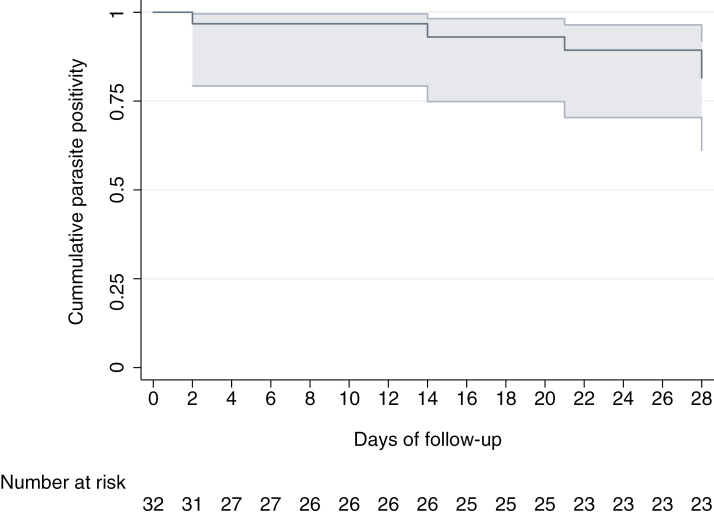
Kaplan–Meier estimates of the cumulative probability of parasite positivity by day 28 measured by quantitative polymerase chain reaction.

**Figure 2.**
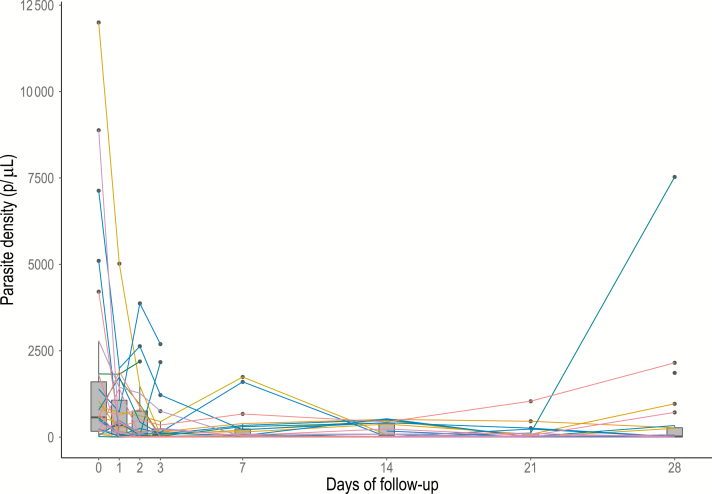
Distributions of individual-level parasite densities observed for each day of follow-up of 32 afebrile *Plasmodium falciparum*–infected individuals over 28 days. 100 parasites/μL represents the threshold below which infections are considered submicroscopic. Abbreviation: p/μL, parasites per microliter.

**Figure 3.**
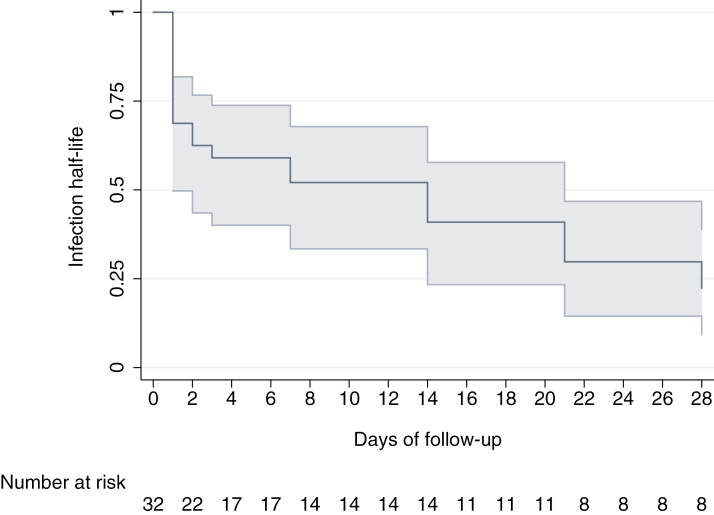
Kaplan–Meier estimates of the cumulative probability of failure to reduce parasitemia levels by at least 50% of their initial value—or half-life probability measured by quantitative polymerase chain reaction.

The cumulative probability of parasite positivity by day 28 based on qPCR diagnosis was 81.5% (95% CI, 61.1–91.9), with only 5 of the 32 individuals becoming negative by qPCR during follow up: 1 on day 2, 1 on day 14, 1 on day 21, and 2 on day 28 (Figure 1). The GMPD of infections as quantified by qPCR dropped by 70% (risk ratio, 0.29; 95% CI, .29–.30) by the end of follow-up (Table 2). The GMPD on day 0 was 516.1 parasites/μL and fell to <100 parasites/μL by day 3, reaching 56.7 parasites/μL on day 28 (*P* < .001) (Figure 2). Ten (31%) individuals successfully reduced parasitemia levels by at least 50% of their initial value within the first day of follow-up (Figure 3), and 5 more did so by the 7th day of follow-up. By the end of the study, the half-life cumulative probability was 22% (95% CI, 9.3–38.9) as the initial parasitemia levels were sustainably halved in 23 of 32 participants. Finally, in 4 of the 32 infections, parasite levels increased during follow-up to levels higher than their initial value (participants 18, 23, 55 and 110 in Supplementary Table 1).

**Table 2.** Parasite Density Dynamics Assessed Through Poisson Regression Random-Effect Models of the Geometric Mean Parasite Density Variations Through Time, and Kaplan–Meier Half-Life Clearance Estimates of the Probability of Failure to Reduce the Initial Parasitemia by Half by Day 28 of Follow-up

Day	Positive by PCR (No.)^a^	Poisson Regression Random-Effect Model		Kaplan–Meier Half-Life Clearance	
		Geometric Mean Parasitemia by qPCR (95% CI)	Risk Ratio (95% CI)	Half-Life Clearance (LFU)	Cumulative Probability of Failure, % (95% CI)
**0**	31 (31)	511.1 (279.1–935.9)	1		
**1**	31 (31)	208.5 (101.0–430.6)	0.41 (.39–.41)	10 (0)	0.69 (.49–.81)
**2**	28 (30)	120.2 (51.3–281.8)	0.31 (.30–.31)	2 (2)	0.63 (.44–.58)
**3**	26 (28)	81.9 (39.8–168.6)	0.18 (.18–.19)	1 (0)	0.59 (.40–.74)
**7**	22 (24)	66.4 (28.5–154.6)	0.14 (.14–.14)	2 (1)	0.52 (.33–.68)
**14**	24 (26)	53.6 (24.2–118.8)	0.09 (.08–.09)	3 (0)	0.41 (.23–.58)
**21**	18 (22)	30.4 (12.2–76.0)	0.05 (.05–.05)	3 (0)	0.30 (.14–.47)
**28**	21 (25)	56.7 (18.3–175.4)	0.29 (.29–.30)	2 (1)	0.22 (.09–.39)

Abbreviations: CI, confidence interval; LFU, lost to follow-up; PCR, polymerase chain reaction; qPCR, quantitative polymerase chain reaction.

^a^Three individuals had missing qPCR data (on days 0, 1, and 21, respectively), and 9 skipped 1 day of follow-up (1 on day 2, 1 on day 3, 4 on day 7, and 3 on day 21).

**Figure 4.**
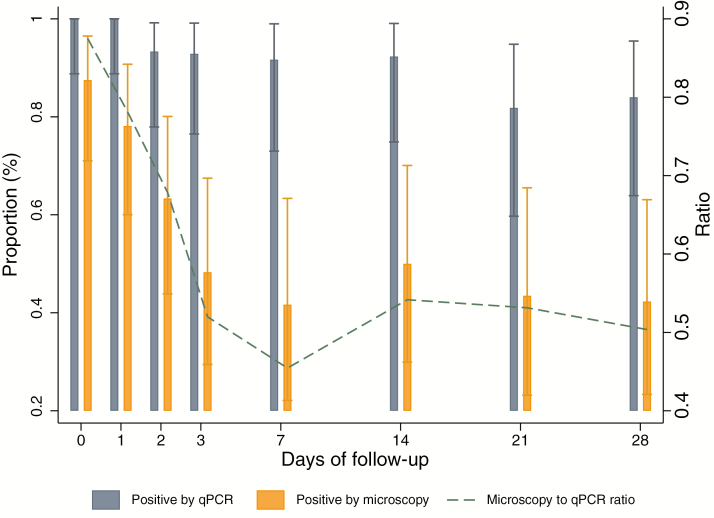
Proportion of positive samples by real-time quantitative polymerase chain reaction and microscopy per follow-up observation (bars, left *y* axis), and ratio of samples detected by polymerase chain reaction and microscopy through time (line, right *y* axis) during the follow-up of 32 afebrile *Plasmodium falciparum*–infected individuals at baseline. Abbreviation: qPCR, quantitative polymerase chain-reaction.

All infections detected by microscopy were also detected by qPCR. The ratio of positive samples by microscopy to qPCR decreased from 0.9 (n = 28/31) on day 0 to 0.52 (n = 11/21) on day 28 (Figure 4). Overall, the GMPD of positive samples by qPCR and microscopy was 351.1 parasites/μL (95% CI, 266.8–462.1), and the parasite density among positive samples undetected by microscopy was 13.8 parasites/μL (95% CI, 9.6–19.8).

The Akaike’s information criterion values obtained after modeling the infection dynamics suggest that the best model fit to the data was obtained assuming a Gompertz distribution (Table 3). The Kolmogorov-Smirnov test results for all distributions showed a modest fit for the 3 distributions. The predicted proportion of individuals who would continue to be infected by day 28 ranged 81%–82% for all distributions, and the estimated median lifetime was 41.6, 61.5, and 73.2 days as estimated by the Gompertz, Weibull, and gamma distributions, respectively (Table 3).

**Table 3.** Estimates of Parameters and Expected Lifetimes, the Akaike Information Criterion, the Kolmogorov-Smirnov Statistics, and the Estimated Proportion of Infected Individuals at Day 28 for the Exponential, Weibull, Log-Normal, and Gamma Distributions

Survival Distribution	Scale	Shape	Median Lifetime	AIC	K-S (*P* value)	% Infected at Day 28
Gompertz	0.084^a^	0.002	41.6	61.5	0.815 (<.001)	81.5%
Weibull	83.5	1.5	61.5	62.9	0.342 (<.001)	82.3%
Gamma	64.2	1.6	73.2	63.1	0.820 (<.001)	82.0%

Abbreviations: AIC, Akaike information criterion; K–S, Kolmogorov–Smirnov.

^a^Rate.

## DISCUSSION

This longitudinal analysis of *P. falciparum*–infected afebrile adults in southern Mozambique followed for 28 days after detection of infection by RDT shows that most study participants remained infected until the end of the study without developing fever and were able to control parasite densities at submicroscopic levels after the first few days of follow-up, following a Gompertz distribution. These findings suggest a rapid decline of potential transmissibility and detectability of recently acquired infections in semi-immune individuals in endemic areas, offering novel information about natural parasite dynamics that can be used to design malaria elimination interventions and model their impact.

Despite not achieving full parasite clearance, most individuals were able to halve their parasite densities within the 28-day follow-up period. In fact, parasite densities drastically decreased during the first 4 days of follow-up to levels below the typical microscopy and RDT detection threshold (100 parasites/μL) and remained below this parasitemia level until the end of the study. Similar patterns have been reported by Bruce-Chwatt et al, who measured daily parasitemia by microscopy after the acquisition of infection among Nigerian adults [16]. A study using an ultrasensitive PCR to monitor parasite densities monthly also showed that asymptomatic infections in adults decreased and remained at levels <100 parasites/μL [18], but there was no up-to-date-evidence of the daily dynamics of infection in adults using molecular techniques. Our findings suggest that individuals from endemic areas, who have acquired some level of antiparasite immunity, are able to rapidly control infections within a few days after detection by RDT without completely clearing the infection by day 28.

Several studies have observed a direct association between asexual parasitemia and gametocyte carriage [33]. In this context, our results suggest that asymptomatic infections in adults have a short period (3–4 days) of detectable parasitemia by microscopy or RDT during which gametocyte carriage and efficiency of transmission to mosquitoes would be expected to be the highest. Nevertheless, these infections seem to rapidly fall and stabilize at low parasitemia levels at which the efficiency of transmission to mosquitoes decreases [34]. Although in a control context this pool of infections could still contribute to transmission due to the abundance in the community [35], in elimination contexts, where the reservoir of asymptomatic infections in the community would decrease along wih the vector density as a result of the interventions deployed, the overall contribution of these remaining infections could decrease.

The ratio of infections detected by microscopy to those detected by qPCR rapidly decreased between day 0 and day 28 from 0.9 to 0.52, suggesting that approximately half of the afebrile infections may not be detected a few days after infection unless a more sensitive diagnostic tool is used. Blood samples that were positive by microscopy and qPCR had a GMPD of 351.1 parasites/μL, compared with 13.8 parasites/μL observed in blood samples that were only positive by qPCR. In addition, we show that afebrile infections that become chronic in semi-immune adults linger below 100 parasites/μL and above 5 parasites/μL based on the qPCR detection threshold.

Mathematical modeling of the data showed the Gompertz distribution as the best fit for *P. falciparum* natural infections, in line with previous reports [31, 32]. This distribution is widely used in demography and survival analysis because of its flexibility. Nevertheless, the estimated duration of infection using the Gompertz distribution to model these data was much lower (41.6 days) than values previously reported in other studies using the same distribution (139.9–209.5 days) [31]. Different immunity levels among individuals included in the datasets used for past models could partly explain these differences. However, the results presented here should be interpreted with caution given the limited fit identified through the Kolmogorov–Smirnov test for all distributions tested, as well as the reduced small sample size and lack of parasite clearance events observed throughout the study period. Similar modeling exercises using data from larger studies that are able to discern between reinfections and recrudescence would be required to fully demonstrate a good fit of any of the distributions evaluated here and to accurately predict duration of infection.

This analysis is subject to a series of limitations that should be considered when interpreting the results. First, the exact time at which each participant acquired the infection could not be assessed. Thus, the survival analysis was performed assuming the same duration of infection since recruitment for each individual at every follow-up time point. A study using microscopy data had previously identified that untreated parasite densities in immune individuals tend to decrease in a short period of time, thus suggesting that infections that could still be identified through an RDT or microscopy could be considered recent and assumed to be of similar duration at the time of recruitment [16]. Our observation of a steep decline in parasitemia a few days after detection of infection by RDT further supports this assumption. However, we could not exclude the possibility that these were older infections detected as a result of parasite density oscillations. Second, the 28 days of follow-up limited our capacity to observe the natural dynamics of *P. falciparum*–untreated infections for a longer period of time. Third, the study did not collect extensive clinical data, such as hemoglobin levels, to assess the adverse impact of these afebrile (albeit not necessarily asymptomatic) chronic infections [2]. Additionally, low parasitemia levels precluded the molecular genotyping of infecting parasites to distinguish recrudescence from new infections [27, 36]. Finally, diurnal variations of body temperature, which may influence the fever estimates depending on the time when the measurement is performed [37], were not assessed in this study.

In conclusion, this study suggests that afebrile *P. falciparum* infections in semi-immune adults tend to transform into submicroscopic infections soon after being detected by RDTs or microscopy. Although the role of these low-density infections in sustaining local transmission is still widely unknown, the association of gametocytemia with parasite biomass suggests a short duration of potentially high transmissibility before reductions in parasite densities [33, 38]. Nevertheless, the infectivity of submicroscopic infections could also depend on the duration of infection, which we have estimated to range between 41.6 and 73.2 days depending on the distribution used, but others have observed to be only a few weeks or as long as a few years [31], or on the parasite density oscillations over time. To contribute to this question, this study also shows that natural infections follow a Gompertz distribution shape, which is crucial for the accurate prediction of infection duration at the community level through mathematical modeling. Mass antimalarial administrations would sharply reduce the asymptomatic reservoir of infection and consequently decrease its contribution to transmission as a group. As more information is gathered about afebrile infections, its relevance in the context of malaria elimination will be revealed, thus allowing policy makers to design and implement strategies that will target the main sources of transmission in the community in the race against this disease.

## Supplementary Data

Supplementary materials are available at *Clinical Infectious Diseases* online. Consisting of data provided by the authors to benefit the reader, the posted materials are not copyedited and are the sole responsibility of the authors, so questions or comments should be addressed to the corresponding author.

Supplementary Material

## Notes

***Acknowledgments.*** We thank Julia Montaña for her editorial assistance on this manuscript. We would also like to thank all of the study participants for their collaboration. We thank everyone who supported this study directly or indirectly through fieldwork or clinical, laboratory or analysis support. Q. B. is an ICREA (Institut Català de la Recerca i Estudis Avançats; Catalan Government) Research Professor. A. M. is supported by the Instituto de Salud Carlos III (CES10/021-I3SNS) and the Departament d’Universitats i Recerca de la Generalitat de Catalunya (AGAUR; 2017SGR664). ISGlobal is a member of the CERCA Programme, Generalitat de Catalunya. Finally, we would also like to thank the Bill and Melinda Gates Foundation for providing the funds for this study.

***Financial support.*** This study was supported by the Bill and Melinda Gates Foundation and Obra Social “*la Caixa*” Partnership for the Elimination of Malaria in Southern Mozambique (OPP1094233).

***Potential conflicts of interest.*** All authors: No reported conflicts of interest. All authors have submitted the ICMJE Form for Disclosure of Potential Conflicts of Interest. Conflicts that the editors consider relevant to the content of the manuscript have been disclosed.
