# Supplementary material for: Dynamics of Afebrile Plasmodium falciparum Infections in Mozambican Men
Source: Clin Infect Dis. 2018 Mar 13;67(7):1045–52. doi: 10.1093/cid/ciy219 (PMC6137111; doi:10.1093/cid/ciy219)
Supplement: Supplementary Material [file ciy219_suppl_supplementary_material.docx]

**Supplementary Table:**

**S. Table 1:** Parasite density levels measured by microscopy and qPCR for each study participant at every follow-up visit. This table also presents the duration of infection and time to infection half-life by qPCR, used to compute Kaplan Meier probabilities. Missing qPCR densities of individuals who were not lost to follow-up are identified with a dot ( . ). Individuals with one missing day of follow-up are identified with a dash ( - ). Cells containing “lfu” specify the day at which individuals were lost to follow-up and censored from the survival analysis, and empty cells represent the following lost to follow-up days.

| Participant ID | Diagnostic  Method | Day  0 | Day 1 | Day 2 | Day 3 | Day 7 | Day 14 | Day 21 | Day 28 | Days of Infection | Time to Infection Half-life |
| --- | --- | --- | --- | --- | --- | --- | --- | --- | --- | --- | --- |
| 3 | Micr | 46 | 0 | 0 | 0 | 0 | 0 | 0 | 0 |  |  |
|  | qPCR | 85 | 1 | 0 | 0 | 45 | 9 | . | 22 | 28 | 14 |
| 9 | Micr | 347 | 53 | 156 | 0 | lfu |  |  |  |  |  |
|  | qPCR | 797 | 639 | 801 | . | lfu |  |  |  | 2 | 2 |
| 15 | Micr | 375 | 114 | 88 | 359 | 287 | 53 | 568 | 55 |  |  |
|  | qPCR | 155 | 326 | 161 | 154 | 392 | 515 | 459 | 272 | 28 | 28 |
| 18 | Micr | 310 | 37 | 12 | 362 | 66 | 249 | 206 | 762 |  |  |
|  | qPCR | 580 | 426 | 13 | 147 | 148 | 376 | 82 | 965 | 28 | 28 |
| 21 | Micr | 15472 | 9502 | 1262 | 102 | 0 | 123 | 0 | 88 |  |  |
|  | qPCR | 12000 | 5020 | 1480 | 70 | 204 | 482 | 2 | 254 | 28 | 1 |
| 23 | Micr | 380 | 588 | 314 | 294 | 347 | 22 | - | 2616 |  |  |
|  | qPCR | 891 | 717 | 616 | 449 | 1740 | 68 | - | 1860 | 28 | 28 |
| 27 | Micr | 398 | 155 | 11 | 0 | - | 0 | 0 | 0 |  |  |
|  | qPCR | 1040 | 168 | 83 | 4 | - | 0 | 0 | 1 | 28 | 1 |
| 30 | Micr | 60 | 0 | 0 | 0 | 9 | lfu |  |  |  |  |
|  | qPCR | 107 | 69 | 23 | 18 | 93 | lfu |  |  | 7 | 7 |
| 32 | Micr | 0 | 11 | 17 | lfu |  |  |  |  |  |  |
|  | qPCR | 25 | . | 37 | lfu |  |  |  |  | 2 | 2 |
| 39 | Micr | 318 | 305 | 447 | 0 | 0 | 0 | 0 | 0 |  |  |
|  | qPCR | . | 1680 | 947 | 32 | 10 | 12 | 14 | 1 | 28 | 28 |
| 40 | Micr | 978 | 782 | 194 | - | 0 | 0 | 16 | 89 |  |  |
|  | qPCR | 1830 | 1820 | 2190 | - | 74 | 2 | 22 | 68 | 28 | 7 |
| 45 | Micr | 0 | 146 | 0 | 0 | 0 | 0 | 0 | 0 |  |  |
|  | qPCR | 15 | 1 | 5 | 32 | 3 | 14 | 0 | 1 | 28 | 21 |
| 46 | Micr | 213 | 44 | lfu |  |  |  |  |  |  |  |
|  | qPCR | 554 | 109 | lfu |  |  |  |  |  | 1 | 1 |
| 55 | Micr | 0 | 319 | 0 | 2242 | - | 0 | 263 | 10567 |  |  |
|  | qPCR | 51 | 417 | 56 | 2170 | - | 5 | 117 | 7530 | 28 | 28 |
| 56 | Micr | 270 | 0 | 28 | 0 | 80 | 104 | - | 0 |  |  |
|  | qPCR | 210 | 39 | 50 | 30 | 303 | 411 | - | 16 | 28 | 28 |
| 57 | Micr | 395 | 640 | 0 | 34 | 39 | 106 | 0 | 1279 |  |  |
|  | qPCR | 781 | 1740 | 437 | 84 | 338 | 503 | 16 | 335 | 28 | 21 |
| 62 | Micr | 857 | 4281 | 2889 | 1791 | 37 | 357 | 100 | 0 |  |  |
|  | qPCR | 7130 | 1990 | 2630 | 1220 | 217 | 417 | 259 | 14 | 28 | 1 |
| 66 | Micr | 454 | 44 | 98 | 36 | 0 | 753 | 0 | 135 |  |  |
|  | qPCR | 504 | 58 | 250 | 118 | 45 | 531 | 5 | 67 | 28 | 21 |
| 70 | Micr | 194 | 0 | - | 0 | 0 | 0 | 0 | 0 |  |  |
|  | qPCR | 241 | 17 | - | 6 | 13 | 28 | 24 | 18 | 28 | 1 |
| 72 | Micr | 7187 | 0 | 38 | 0 | 1262 | 0 | 3031 | 0 |  |  |
|  | qPCR | 5100 | 78 | 86 | 138 | 1600 | 11 | 233 | 11 | 28 | 1 |
| 78 | Micr | 1634 | 2653 | 5737 | 5681 | - | 33 | 0 | 0 |  |  |
|  | qPCR | 1390 | 726 | 3870 | 2690 | - | 176 | 2 | 6 | 28 | 14 |
| 81 | Micr | 180 | 329 | 593 | 0 | - | 0 | 0 | 0 |  |  |
|  | qPCR | 39 | 481 | 124 | 232 | - | 6 | 0 | 0 | 21 | 14 |
| 84 | Micr | 0 | 236 | 0 | 48 | 19 | 12 | 0 | 0 |  |  |
|  | qPCR | 182 | 137 | 83 | 253 | 34 | 85 | 2 | 0 | 28 | 7 |
| 92 | Micr | 11842 | 4625 | 2902 | 2399 | 0 | 0 | 0 | 0 |  |  |
|  | qPCR | 2790 | 1440 | 1270 | 752 | 14 | 9 | 2 |  | 21 | 2 |
| 101 | Micr | 11091 | 0 | 0 | 0 | 0 | 0 | 20 | 362 |  |  |
|  | qPCR | 8880 | 144 | 0 | 5 | 87 | 89 | 105 | 10 | 28 | 1 |
| 104 | Micr | 566 | 1378 | 0 | 0 | 0 | 0 | 0 | 0 |  |  |
|  | qPCR | 159 | 1410 | 11 | 22 | 1 | 0 | 0 | 0 | 14 | 2 |
| 108 | Micr | 2578 | 2649 | 18 | 79 | 0 | 36 | 16 | 0 |  |  |
|  | qPCR | 431 | 126 | 6 | 29 | 0 | 30 | 36 | 63 | 28 | 1 |
| 110 | Micr | 375 | 961 | 0 | 238 | 0 | 103 | 211 | 222 |  |  |
|  | qPCR | 179 | 428 | 116 | 220 | 48 | 237 | 45 | 716 | 28 | 28 |
| 114 | Micr | 411 | 0 | 0 | 0 | 0 | lfu |  |  |  |  |
|  | qPCR | 682 | 11 | 0 | 0 | 0 | lfu |  |  | 2 | 1 |
| 115 | Micr | 1067 | 44 | 0 | 0 | lfu |  |  |  |  |  |
|  | qPCR | 1810 | 19 | 4 | 5 | lfu |  |  |  | 3 | 1 |
| 120 | Micr | 3602 | 4012 | 854 | 612 | 1215 | 617 | 890 | 10337 |  |  |
|  | qPCR | 4210 | 273 | 503 | 350 | 670 | 440 | 1040 | 2150 | 28 | 28 |
| 124 | Micr | 1305 | 4805 | 4574 | 0 | 0 | 0 | - | 0 |  |  |
|  | qPCR | 572 | 1900 | 866 | 12 | 2 | 1 | - | 0 | 28 | 3 |
